# Supplementary material for: Patlak Slope versus Standardized Uptake Value Image Quality in an Oncologic PET/CT Population: A Prospective Cross-Sectional Study
Source: Diagnostics (Basel). 2024 Apr 24;14(9):883. doi: 10.3390/diagnostics14090883 (PMC11083784; doi:10.3390/diagnostics14090883)
Supplement: Supplementary file 1 [file diagnostics-14-00883-s001.zip › diagnostics-2931762-SI.pdf]

Supplemental Table S1. Two-reader overall image quality assessments.

Variable 1 (V1)	V1 Mean (Min, Max)	Variable 2 (V2)	V2 Mean (Min, Max)	*P* value (V1 vs. V2)	*V1 > V2*	*V1 = V2*	*V1 < V2*	*% agreement (95% CI)*
**All tracers: scale 1-4 (1 = worst; 4 = best)**								
SUV-early		*PS-early*						
R1	3.88 (2, 4)	R1	1.19 (1, 2)	<0.001	R1: 43	R1: 0	R1: 0	93.0
R2	3.84 (3, 4)	R2	2.14 (1, 4)	<0.001	R2: 40	R2: 3	R2: 0	(80.9, 98.5)
SUV-late		*PS-late*						
R1	2.79 (1, 3)	R1	3.95 (3, 4)	<0.001	R1: 0	R1: 1	R1: 42	32.6
R2	3.44 (1, 4)	R2	3.95 (3, 4)	0.002	R2: 2	R2: 26	R2: 15	(19.0, 48.5)
SUV-early		*SUV-late*						
R1	3.88 (2, 4)	R1	2.79 (1, 3)	<0.001	R1: 41	R1: 1	R1: 1	32.6
R2	3.84 (3, 4)	R2	3.44 (1, 4)	0.03	R2: 14	R2: 23	R2: 6	(19.0, 48.5)
PS-early		*PS-late*						
R1	1.19 (1, 2)	R1	3.95 (3, 4)	<0.001	R1: 0	R1: 0	R1: 43	90.7
R2	2.14 (1, 4)	R2	3.95 (3, 4)	<0.001	R2: 0	R2: 3	R2: 40	(77.9, 97.4)
SUV-early		*PS-late*						
R1	3.88 (2, 4)	R1	3.95 (3, 4)	N/A	R1: 1	R1: 39	R1: 3	81.4
R2	3.84 (3, 4)	R2	3.95 (3, 4)	>0.05*	R2: 2	R2: 34	R2: 7	(66.6, 91.6)
PS-early		*SUV-late*						
R1	1.19 (1, 2)	R1	2.79 (1, 3)	<0.001	R1: 0	R1: 2	R1: 41	81.4
R2	2.14 (1, 4)	R2	3.44 (1, 4)	<0.001	R2: 4	R2: 3	R2: 36	(66.6, 91.6)
**[18F]FDG only: scale 1-4 (1 = worst; 4 = best)**								
SUV-early		*PS-early*						
R1	3.94 (3, 4)	R1	1.12 (1, 2)	<0.001	R1: 33	R1: 0	R1: 0	100.0
R2	3.82 (3, 4)	R2	1.94 (1, 3)	<0.001	R2: 33	R2: 0	R2: 0	(89.4, 100.0)
SUV-late		*PS-late*						
R1	2.91 (2, 3)	R1	3.97 (3, 4)	<0.001	R1: 0	R1: 1	R1: 32	18.2
R2	3.82 (3, 4)	R2	3.94 (3, 4)	>0.05*	R2: 2	R2: 25	R2: 6	(7.0, 35.4)
SUV-early		*SUV-late*						
R1	3.94 (3, 4)	R1	2.91 (2, 3)	<0.001	R1: 32	R1: 1	R1: 0	18.2
R2	3.82 (3, 4)	R2	3.82 (3, 4)	>0.05*	R2: 6	R2: 21	R2: 6	(7.0, 35.4)
PS-early		*PS-late*						
R1	1.12 (1, 2)	R1	3.97 (3, 4)	<0.001	R1: 0	R1: 0	R1: 33	97.0
R2	1.94 (1, 3)	R2	3.94 (3, 4)	<0.001	R2: 0	R2: 1	R2: 32	(84.2, 99.9)
SUV-early		*PS-late*						
R1	3.94 (3, 4)	R1	3.97 (3, 4)	N/A	R1: 1	R1: 30	R1: 2	78.8
R2	3.82 (3, 4)	R2	3.94 (3, 4)	>0.05*	R2: 2	R2: 25	R2: 6	(61.1, 91.0)
PS-early		*SUV-late*						
R1	1.12 (1, 2)	R1	2.91 (2, 3)	<0.001	R1: 0	R1: 0	R1: 33	100.0
R2	1.94 (1, 3)	R2	3.82 (3, 4)	<0.001	R2: 0	R2: 0	R2: 33	(89.4, 100.0)
**DOTATATE only: scale of 1-4 (1 = worst; 4 = best)**								
SUV-early		*PS-early*						
R1	3.63 (2, 4)	R1	1.38 (1, 2)	<0.05*	R1: 8	R1: 0	R1: 0	62.5
R2	3.88 (3, 4)	R2	3.00 (1, 4)	N/A	R2: 5	R2: 3	R2: 0	(24.5, 91.5)
SUV-late		*PS-late*						
R1	2.25 (1, 3)	R1	3.88 (3, 4)	<0.05*	R1: 0	R1: 0	R1: 8	87.5
R2	2.00 (1, 4)	R2	4.00 (4, 4)	<0.05*	R2: 0	R2: 1	R2: 7	(47.3, 99.7)
SUV-early		*SUV-late*						
R1	3.63 (2, 4)	R1	2.25 (1, 3)	<0.05*	R1: 7	R1: 0	R1: 1	75.0
R2	3.88 (3, 4)	R2	2.00 (1, 4)	<0.05*	R2: 6	R2: 2	R2: 0	(34.9, 96.8)
PS-early		*PS-late*						
R1	1.38 (1, 2)	R1	3.88 (3, 4)	<0.05*	R1: 0	R1: 0	R1: 8	75.0
R2	3.00 (1, 4)	R2	4.00 (4, 4)	<0.05*	R2: 0	R2: 2	R2: 6	(34.9, 96.8)
SUV-early		*PS-late*						
R1	3.63 (2, 4)	R1	3.88 (3, 4)	N/A	R1: 0	R1: 7	R1: 1	100.0
R2	3.88 (3, 4)	R2	4.00 (4, 4)	N/A	R2: 0	R2: 7	R2: 1	(63.0, 100.0)
PS-early		*SUV-late*						
R1	1.38 (1, 2)	R1	2.25 (1, 3)	<0.05*	R1: 0	R1: 2	R1: 6	12.5
R2	3.00 (1, 4)	R2	2.00 (1, 4)	N/A	R2: 4	R2: 3	R2: 1	(0.3, 52.6)

Abbreviations: Max = maximum; Min = minimum; R1 = reader 1; R2 = reader 2

* Insufficient *n* for precise *P* value calculation but still possible to determine statistical significance

N/A = insufficient cases with different values for V1 and V2 to calculate a *P* value

Note. – The V1 > V2, V1 = V2, and V1 < V2 columns indicate the number of case in which each reader assigned higher, equal, and lower scores to variable 1 relative to variable 2, respectively.

Supplemental Table S2. Two-reader image noise assessments.

Variable 1 (V1)	V1 Mean (Min, Max)	Variable 2 (V2)	V2 Mean (Min, Max)	*P* value (V1 vs. V2)	*V1 > V2*	*V1 = V2*	*V1 < V2*	*% agreement (95% CI)*
**All tracers: scale 1-4 (1 = worst; 4 = best)**								
SUV-early		*PS-early*						
R1	3.86 (2, 4)	R1	1.14 (1, 2)	<0.001	R1: 43	R1: 0	R1: 0	95.3
R2	3.84 (2, 4)	R2	1.74 (1, 4)	<0.001	R2: 41	R2: 2	R2: 0	(84.1, 99.4)
SUV-late		*PS-late*						
R1	2.63 (1, 3)	R1	3.95 (3, 4)	<0.001	R1: 0	R1: 1	R1: 42	60.5
R2	3.14 (1, 4)	R2	3.98 (3, 4)	<0.001	R2: 0	R2: 17	R2: 26	(44.4, 75.0)
SUV-early		*SUV-late*						
R1	3.86 (2, 4)	R1	2.63 (1, 3)	<0.001	R1: 40	R1: 3	R1: 0	55.8
R2	3.84 (2, 4)	R2	3.14 (1, 4)	<0.001	R2: 26	R2: 11	R2: 6	(39.9, 71.0)
PS-early		*PS-late*						
R1	1.14 (1, 2)	R1	3.95 (3, 4)	<0.001	R1: 0	R1: 0	R1: 43	95.3
R2	1.74 (1, 4)	R2	3.98 (3, 4)	<0.001	R2: 0	R2: 1	R2: 42	(84.1, 99.4)
SUV-early		*PS-late*						
R1	3.86 (2, 4)	R1	3.95 (3, 4)	N/A	R1: 1	R1: 38	R1: 4	79.1
R2	3.84 (2, 4)	R2	3.98 (3, 4)	>0.05*	R2: 1	R2: 36	R2: 6	(64.0, 90.0)
PS-early		*SUV-late*						
R1	1.14 (1, 2)	R1	2.63 (1, 3)	<0.001	R1: 1	R1: 4	R1: 38	79.1
R2	1.74 (1, 4)	R2	3.14 (1, 4)	<0.001	R2: 4	R2: 4	R2: 35	(64.0, 90.0)
**[^{18}F]FDG only: scale 1-4 (1 = worst; 4 = best)**								
SUV-early		*PS-early*						
R1	3.91 (3, 4)	R1	1.03 (1, 2)	<0.001	R1: 33	R1: 0	R1: 0	100.0
R2	3.85 (3, 4)	R2	1.58 (1, 3)	<0.001	R2: 33	R2: 0	R2: 0	(89.4, 100.0)
SUV-late		*PS-late*						
R1	2.82 (2, 3)	R1	3.97 (3, 4)	<0.001	R1: 0	R1: 1	R1: 32	54.5
R2	3.45 (3, 4)	R2	3.97 (3, 4)	<0.001	R2: 0	R2: 16	R2: 17	(36.3, 71.9)
SUV-early		*SUV-late*						
R1	3.91 (3, 4)	R1	2.82 (2, 3)	<0.001	R1: 31	R1: 2	R1: 0	48.5
R2	3.85 (3, 4)	R2	3.45 (3, 4)	0.02	R2: 18	R2: 10	R2: 5	(31.0, 66.4)
PS-early		*PS-late*						
R1	1.03 (1, 2)	R1	3.97 (3, 4)	<0.001	R1: 0	R1: 0	R1: 33	100.0
R2	1.58 (1, 3)	R2	3.97 (3, 4)	<0.001	R2: 0	R2: 0	R2: 33	(89.4, 100.0)
SUV-early		*PS-late*						
R1	3.91 (3, 4)	R1	3.97 (3, 4)	N/A	R1: 1	R1: 29	R1: 3	75.8
R2	3.85 (3, 4)	R2	3.97 (3, 4)	>0.05*	R2: 1	R2: 27	R2: 5	(57.8, 89.0)
PS-early		*SUV-late*						
R1	1.03 (1, 2)	R1	2.82 (2, 3)	<0.001	R1: 0	R1: 0	R1: 33	94.0
R2	1.58 (1, 3)	R2	3.45 (3, 4)	<0.001	R2: 0	R2: 2	R2: 31	(79.8, 99.2)
**DOTATATE only: scale of 1-4 (1 = worst; 4 = best)**								
SUV-early		*PS-early*						
R1	3.63 (2, 4)	R1	1.50 (1, 2)	<0.05*	R1: 8	R1: 0	R1: 0	75.0
R2	3.75 (2, 4)	R2	2.63 (1, 4)	<0.05*	R2:6	R2: 2	R2: 0	(34.9, 96.8)
SUV-late		*PS-late*						
R1	1.75 (1, 2)	R1	3.88 (3, 4)	<0.05*	R1: 0	R1: 0	R1: 8	87.5
R2	2.00 (1, 4)	R2	4.00 (4, 4)	<0.05*	R2: 0	R2: 1	R2: 7	(47.3, 99.7)
SUV-early		*SUV-late*						
R1	3.63 (2, 4)	R1	1.75 (1, 2)	<0.05*	R1: 7	R1: 1	R1: 0	75.0
R2	3.75 (2, 4)	R2	2.00 (1, 4)	<0.05*	R2: 6	R2: 1	R2: 1	(34.9, 96.8)
PS-early		*PS-late*						
R1	1.50 (1, 2)	R1	3.88 (3, 4)	<0.05*	R1: 0	R1: 0	R1: 8	87.5
R2	2.63 (1, 4)	R2	4.00 (4, 4)	<0.05*	R2: 0	R2: 1	R2: 7	(47.3, 99.7)
SUV-early		*PS-late*						
R1	3.63 (2, 4)	R1	3.88 (3, 4)	N/A	R1: 0	R1: 7	R1: 1	100.0
R2	3.75 (2, 4)	R2	4.00 (4, 4)	N/A	R2: 0	R2: 7	R2: 1	(63.0, 100.0)
PS-early		*SUV-late*						
R1	1.50 (1, 2)	R1	1.75 (1, 2)	N/A	R1: 1	R1: 4	R1: 3	25.0
R2	2.63 (1, 4)	R2	2.00 (1, 4)	>0.05*	R2: 4	R2: 2	R2: 2	(3.2, 65.0)

Abbreviations: Max = maximum; Min = minimum; R1 = reader 1; R2 = reader 2

* Insufficient *n* for precise *P* value calculation but still possible to determine statistical significance

N/A = insufficient cases with different values for V1 and V2 to calculate a *P* value

Note. – The V1 > V2, V1 = V2, and V1 < V2 columns indicate the number of case in which each reader assigned higher, equal, and lower scores to variable 1 relative to variable 2, respectively.

Supplemental Table S3. Two-reader artifact freeness assessments.

Variable 1 (V1)	V1 Mean (Min, Max)	Variable 2 (V2)	V2 Mean (Min, Max)	*P* value (V1 vs. V2)	*V1 > V2*	*V1 = V2*	*V1 < V2*	*% agreement (95% CI)*
**All tracers: scale 1-4 (1 = worst; 4 = best)**								
SUV-early		*PS-early*						
R1	3.95 (3, 4)	R1	1.14 (1, 2)	<0.001	R1: 43	R1: 0	R1: 0	86.0
R2	3.60 (1, 4)	R2	1.77 (1, 4)	<0.001	R2: 37	R2: 6	R2: 0	(72.0, 94.7)
SUV-late		*PS-late*						
R1	2.84 (1, 4)	R1	3.95 (3, 4)	<0.001	R1: 0	R1: 2	R1: 41	34.9
R2	3.44 (1, 4)	R2	3.98 (3, 4)	0.001	R2: 1	R2: 27	R2: 15	(21.0, 50.1)
SUV-early		*SUV-late*						
R1	3.95 (3, 4)	R1	2.84 (1, 4)	<0.001	R1: 42	R1: 1	R1: 0	34.9
R2	3.60 (1, 4)	R2	3.44 (1, 4)	>0.05*	R2: 14	R2: 18	R2: 11	(21.0, 50.1)
PS-early		*PS-late*						
R1	1.14 (1, 2)	R1	3.95 (3, 4)	<0.001	R1: 0	R1: 0	R1: 43	93.0
R2	1.77 (1, 4)	R2	3.98 (3, 4)	<0.001	R2: 0	R2: 2	R2: 41	(80.9, 98.5)
SUV-early		*PS-late*						
R1	3.95 (3, 4)	R1	3.95 (3, 4)	N/A	R1: 1	R1: 41	R1: 1	72.1
R2	3.60 (1, 4)	R2	3.98 (3, 4)	0.005	R2: 1	R2: 30	R2: 12	(56.3, 84.7)
PS-early		*SUV-late*						
R1	1.14 (1, 2)	R1	2.84 (1, 4)	<0.001	R1: 0	R1: 5	R1: 38	83.7
R2	1.77 (1, 4)	R2	3.44 (1, 4)	<0.001	R2: 4	R2: 3	R2: 36	(69.3, 93.1)
**[^{18}F]FDG only: scale 1-4 (1 = worst; 4 = best)**								
SUV-early		*PS-early*						
R1	4.00 (4, 4)	R1	1.03 (1, 2)	<0.001	R1: 33	R1: 0	R1: 0	87.9
R2	3.52 (1, 4)	R2	1.58 (1, 3)	<0.001	R2: 29	R2: 4	R2: 0	(71.8, 96.6)
SUV-late		*PS-late*						
R1	3.03 (3, 4)	R1	3.97 (3, 4)	<0.001	R1:0	R1: 2	R1: 31	21.2
R2	3.82 (3, 4)	R2	3.97 (3, 4)	>0.05*	R2: 1	R2: 26	R2: 6	(9.0, 39.0)
SUV-early		*SUV-late*						
R1	4.00 (4, 4)	R1	3.03 (3, 4)	<0.001	R1: 32	R1: 1	R1: 0	21.2
R2	3.52 (1, 4)	R2	3.82 (3, 4)	0.10	R2: 6	R2: 16	R2: 11	(9.0, 39.0)
PS-early		*PS-late*						
R1	1.03 (1, 2)	R1	3.97 (3, 4)	<0.001	R1: 0	R1: 0	R1: 33	97.0
R2	1.58 (1, 3)	R2	3.97 (3, 4)	<0.001	R2: 0	R2: 1	R2: 32	(84.2, 99.9)
SUV-early		*PS-late*						
R1	4.00 (4, 4)	R1	3.97 (3, 4)	N/A	R1: 1	R1: 32	R1: 0	66.7
R2	3.52 (1, 4)	R2	3.97 (3, 4)	0.006	R2: 1	R2: 21	R2: 11	(48.1, 82.0)
PS-early		*SUV-late*						
R1	1.03 (1, 2)	R1	3.03 (3, 4)	<0.001	R1: 0	R1: 0	R1: 33	100.0
R2	1.58 (1, 3)	R2	3.82 (3, 4)	<0.001	R2: 0	R2: 0	R2: 33	(89.4, 100.0)
**DOTATATE only: scale of 1-4 (1 = worst; 4 = best)**								
SUV-early		*PS-early*						
R1	3.75 (3, 4)	R1	1.63 (1, 2)	<0.05*	R1: 8	R1: 0	R1: 0	75.0
R2	3.88 (3, 4)	R2	2.75 (1, 4)	<0.05*	R2: 6	R2: 2	R2: 0	(34.9, 96.8)
SUV-late		*PS-late*						
R1	2.00 (1, 3)	R1	3.88 (3, 4)	<0.05*	R1: 0	R1: 0	R1: 8	87.5
R2	2.00 (1, 4)	R2	4.00 (4, 4)	<0.05*	R2: 0	R2: 1	R2: 7	(47.3, 99.7)
SUV-early		*SUV-late*						
R1	3.75 (3, 4)	R1	2.00 (1, 3)	<0.05*	R1: 8	R1: 0	R1: 0	75.0
R2	3.88 (3, 4)	R2	2.00 (1, 4)	<0.05*	R2: 6	R2: 2	R2: 0	(34.9, 96.8)
PS-early		*PS-late*						
R1	1.63 (1, 2)	R1	3.88 (3, 4)	<0.05*	R1: 0	R1: 0	R1: 8	87.5
R2	2.75 (1, 4)	R2	4.00 (4, 4)	<0.05*	R2: 0	R2: 1	R2: 7	(47.3, 99.7)
SUV-early		*PS-late*						
R1	3.75 (3, 4)	R1	3.88 (3, 4)	N/A	R1: 0	R1: 7	R1: 1	100.0
R2	3.88 (3, 4)	R2	4.00 (4, 4)	N/A	R2: 0	R2: 7	R2: 1	(63.0, 100.0)
PS-early		*SUV-late*						
R1	1.63 (1, 2)	R1	2.00 (1, 3)	N/A	R1: 0	R1: 5	R1: 3	25.0
R2	2.75 (1, 4)	R2	2.00 (1, 4)	N/A	R2: 4	R2: 3	R2: 1	(3.19, 65.0)

Abbreviations: Max = maximum; Min = minimum; R1 = reader 1; R2 = reader 2

* Insufficient *n* for precise *P* value calculation but still possible to determine statistical significance

N/A = insufficient cases with different values for V1 and V2 to calculate a *P* value

Note. – The V1 > V2, V1 = V2, and V1 < V2 columns indicate the number of case in which each reader assigned higher, equal, and lower scores to variable 1 relative to variable 2, respectively.

Supplemental Table S4. Two-reader lesion conspicuity assessments.

Variable 1 (V1)	V1 Mean (Min, Max)	Variable 2 (V2)	V2 Mean (Min, Max)	*P* value (V1 vs. V2)	*V1 > V2*	*V1 = V2*	*V1 < V2*	*% agreement (95% CI)*
**All tracers: scale 1-4 (1 = worst; 4 = best)**								
SUV-early		*PS-early*						
R1	2.84 (2, 4)	R1	2.28 (1, 4)	0.003	R1: 23	R1: 15	R1: 5	44.2
R2	3.28 (1, 4)	R2	2.30 (1, 4)	<0.001	R2: 27	R2: 12	R2: 4	(29.1, 60.1)
SUV-late		*PS-late*						
R1	3.53 (1, 4)	R1	3.49 (2, 4)	0.84	R1: 15	R1: 17	R1: 11	39.5
R2	3.58 (1, 4)	R2	3.77 (1, 4)	>0.05*	R2: 5	R2: 29	R2: 9	(25.0, 55.6)
SUV-early		*SUV-late*						
R1	2.84 (2, 4)	R1	3.53 (1, 4)	<0.001	R1: 6	R1: 9	R1: 28	58.1
R2	3.28 (1, 4)	R2	3.58 (1, 4)	>0.05*	R2: 9	R2: 18	R2: 16	(42.1, 73,0)
PS-early		*PS-late*						
R1	2.28 (1, 4)	R1	3.49 (2, 4)	<0.001	R1: 4	R1: 5	R1: 34	69.8
R2	2.30 (1, 4)	R2	3.77 (1, 4)	<0.001	R2: 2	R2: 7	R2: 34	(53.9, 82.8)
SUV-early		*PS-late*						
R1	2.84 (2, 4)	R1	3.49 (2, 4)	<0.001	R1: 7	R1: 9	R1: 27	41.9
R2	3.28 (1, 4)	R2	3.77 (1, 4)	0.006	R2: 2	R2: 25	R2: 16	(27.0, 57.9)
PS-early		*SUV-late*						
R1	2.28 (1, 4)	R1	3.53 (1, 4)	<0.001	R1: 4	R1: 7	R1: 32	65.1
R2	2.30 (1, 4)	R2	3.58 (1, 4)	<0.001	R2: 4	R2: 8	R2: 31	(49.1, 79.0)
**[^{18}F]FDG only: scale 1-4 (1 = worst; 4 = best)**								
SUV-early		*PS-early*						
R1	2.64 (2, 4)	R1	2.12 (1, 4)	0.02	R1: 16	R1: 13	R1: 4	39.4
R2	3.09 (1, 4)	R2	2.15 (1, 4)	<0.001	R2: 20	R2: 9	R2: 4	(23.0, 57.9)
SUV-late		*PS-late*	–					
R1	3.73 (3, 4)	R1	3.58 (2, 4)	0.32	R1: 11	R1: 15	R1: 7	33.3
R2	3.88 (2, 4)	R2	3.73 (1, 4)	>0.05*	R2: 5	R2: 26	R2: 2	(18.0, 51.8)
SUV-early		*SUV-late*						
R1	2.64 (2, 4)	R1	3.73 (3, 4)	<0.001	R1: 1	R1: 5	R1: 27	51.5
R2	3.09 (1, 4)	R2	3.88 (2, 4)	0.001	R2: 2	R2: 15	R2: 16	(33.5, 69.2)
PS-early		*PS-late*						
R1	2.12 (1, 4)	R1	3.58 (2, 4)	<0.001	R1: 2	R1: 2	R1: 29	69.7
R2	2.15 (1, 4)	R2	3.73 (1, 4)	<0.001	R2: 2	R2: 4	R2: 27	(51.2, 84.4)
SUV-early		*PS-late*						
R1	2.64 (2, 4)	R1	3.58 (2, 4)	<0.001	R1: 1	R1: 8	R1: 24	48.5
R2	3.09 (1, 4)	R2	3.73 (1, 4)	0.006	R2: 2	R2: 15	R2: 16	(30.8, 66.4)
PS-early		*SUV-late*						
R1	2.12 (1, 4)	R1	3.73 (3, 4)	<0.001	R1: 1	R1: 4	R1: 28	69.7
R2	2.15 (1, 4)	R2	3.88 (2, 4)	<0.001	R2: 0	R2: 5	R2: 28	(51.2, 84.4)
**DOTATATE only: scale of 1-4 (1 = worst; 4 = best)**								
SUV-early		*PS-early*						
R1	3.50 (2, 4)	R1	3.00 (2, 4)	>0.05*	R1: 5	R1: 2	R1: 1	50.0
R2	3.88 (3, 4)	R2	3.13 (2, 4)	N/A	R2: 5	R2: 3	R2: 0	(15.7, 84.3)
SUV-late		*PS-late*						
R1	2.63 (1, 4)	R1	3.38 (2, 4)	>0.05*	R1: 2	R1: 2	R1: 4	62.5
R2	2.38 (1, 4)	R2	3.88 (3, 4)	<0.05*	R2: 0	R2: 2	R2: 6	(24.5, 91.5)
SUV-early		*SUV-late*						
R1	3.50 (2, 4)	R1	2.63 (1, 4)	N/A	R1: 5	R1: 3	R1: 0	87.5
R2	3.88 (3, 4)	R2	2.38 (1, 4)	<0.05*	R2: 6	R2: 2	R2: 0	(47.3, 99.7)
PS-early		*PS-late*						
R1	3.00 (2, 4)	R1	3.38 (2, 4)	>0.05*	R1: 2	R1: 2	R1: 4	75.0
R2	3.13 (2, 4)	R2	3.88 (3, 4)	N/A	R2: 0	R2: 3	R2: 5	(34.9, 96.8)
SUV-early		*PS-late*						
R1	3.50 (2, 4)	R1	3.38 (2, 4)	>0.05*	R1: 4	R1: 1	R1: 3	12.5
R2	3.88 (3, 4)	R2	3.88 (3, 4)	N/A	R2: 0	R2: 8	R2: 0	(0.3, 52.6)
PS-early		*SUV-late*						
R1	3.00 (2, 4)	R1	2.63 (1, 4)	N/A	R1: 3	R1: 3	R1: 2	50.0
R2	3.13 (2, 4)	R2	2.38 (1, 4)	N/A	R2: 4	R2: 3	R2: 1	(15.7, 84.3)

Abbreviations: Max = maximum; Min = minimum; R1 = reader 1; R2 = reader 2

* Insufficient *n* for precise *P* value calculation but still possible to determine statistical significance

N/A = insufficient cases with different values for V1 and V2 to calculate a *P* value

Note. – The V1 > V2, V1 = V2, and V1 < V2 columns indicate the number of case in which each reader assigned higher, equal, and lower scores to variable 1 relative to variable 2, respectively.

Supplemental Table S5. Two-reader relative lesion number assessments.

Variable 1 (V1)	V1 Mean (Min, Max)	Variable 2 (V2)	V2 Mean (Min, Max)	*P* value (V1 vs. V2)	*V1 > V2*	*V1 = V2*	*V1 < V2*	*% agreement (95% CI)*
**All tracers: scale 1-4 (1 = worst; 4 = best)**								
SUV-early		*PS-early*						
R1	0.77 (0, 10)	R1	0.23 (0, 2)	0.10	R1: 10	R1: 27	R1: 6	58.1
R2	0.58 (0, 8)	R2	0.12 (0, 3)	0.02	R2: 10	R2: 31	R2: 2	(42.1, 73.0)
SUV-late		*PS-late*						
R1	2.09 (0, 25)	R1	1.35 (0, 11)	0.04	R1: 10	R1: 30	R1: 3	65.1
R2	1.12 (0, 8)	R2	1.05 (0, 7)	N/A	R2: 2	R2: 38	R2: 3	(49.1, 79.0)
SUV-early		*SUV-late*						
R1	0.77 (0, 10)	R1	2.09 (0, 25)	<0.001	R1: 4	R1: 21	R1: 18	65.1
R2	0.58 (0, 8)	R2	1.12 (0, 8)	0.04	R2: 2	R2: 28	R2: 13	(49.1, 79.0)
PS-early		*PS-late*						
R1	0.23 (0, 2)	R1	1.35 (0, 11)	<0.001	R1: 2	R1: 22	R1: 19	65.1
R2	0.12 (0, 3)	R2	1.05 (0, 7)	<0.001	R2: 0	R2: 26	R2: 17	(49.1, 79.0)
SUV-early		*PS-late*						
R1	0.77 (0, 10)	R1	1.35 (0, 11)	0.006	R1: 2	R1: 27	R1: 14	65.1
R2	0.58 (0, 8)	R2	1.05 (0, 7)	0.02	R2: 1	R2: 29	R2: 13	(49.1, 79.0)
PS-early		*SUV-late*						
R1	0.23 (0, 2)	R1	2.09 (0, 25)	<0.001	R1: 2	R1: 21	R1: 20	65.1
R2	0.12 (0, 3)	R2	1.12 (0, 8)	0.001	R2: 2	R2: 23	R2: 18	(49.1, 79.0)
**$[^{18}F]$FDG only: scale 1-4 (1 = worst; 4 = best)**								
SUV-early		*PS-early*						
R1	0.64 (0, 10)	*R1*	0.24 (0, 2)	0.48	R1: 6	R1: 21	R1: 6	57.6
R2	0.58 (0, 8)	*R2*	0.06 (0, 1)	0.04	R2: 9	R2: 22	R2: 2	(39.2, 74.5)
SUV-late		*PS-late*						
R1	2.42 (0, 25)	*R1*	1.45 (0, 11)	<0.05*	R1: 7	R1: 26	R1: 0	69.7
R2	1.42 (0, 8)	*R2*	1.15 (0, 7)	N/A	R2: 2	R2: 30	R2: 1	(51.2, 84.4)
SUV-early		*SUV-late*						
R1	0.64 (0, 10)	*R1*	2.42 (0, 25)	<0.001	R1: 0	R1: 16	R1: 17	72.7
R2	0.58 (0, 8)	*R2*	1.42 (0, 8)	0.002	R2: 0	R2: 21	R2: 12	(54.5, 86.7)
PS-early		*PS-late*						
R1	*0.24 (0, 2)*	R1	1.45 (0, 11)	0.001	R1: 2	R1: 15	R1: 16	66.7
R2	*0.06 (0, 1)*	R2	1.15 (0, 7)	<0.001	R2: 0	R2: 18	R2: 15	(48.1, 82.0)
SUV-early		*PS-late*						
R1	0.64 (0, 10)	*R1*	1.45 (0, 11)	<0.001	R1: 0	R1: 20	R1: 13	60.6
R2	0.58 (0, 8)	*R2*	1.15 (0, 7)	0.02	R2: 1	R2: 20	R2: 12	(42.1, 77.0)
PS-early		*SUV-late*						
R1	*0.24 (0, 2)*	*R1*	2.42 (0, 25)	<0.001	R1: 0	R1: 16	R1: 17	72.7
R2	*0.06 (0, 1)*	*R2*	1.42 (0, 8)	<0.001	R2: 1	R2: 15	R2: 17	(57.5, 87.9)
**DOTATATE only: scale of 1-4 (1 = worst; 4 = best)**								
SUV-early		*PS-early*						
R1	1.25 (0, 5)	R1	0.25 (0, 1)	N/A	R1: 3	R1: 5	R1: 0	50.0
R2	0.75 (0, 3)	R2	0.38 (0, 3)	N/A	R2: 1	R2: 7	R2: 0	(15.7, 84.3)
SUV-late		*PS-late*						
R1	1.00 (0, 7)	R1	1.13 (0, 6)	N/A	R1: 2	R1: 3	R1: 3	37.5
R2	0.00 (0, 0)	R2	0.75 (0, 3)	N/A	R2: 0	R2: 6	R2: 2	(8.5, 75.5)
SUV-early		*SUV-late*						
R1	1.25 (0, 5)	R1	1.00 (0, 7)	N/A	R1: 4	R1: 3	R1: 1	37.5
R2	0.75 (0, 3)	R2	0.00 (0, 0)	N/A	R2: 2	R2: 6	R2: 0	(8.5, 75.5)
PS-early		*PS-late*						
R1	0.25 (0, 1)	R1	1.13 (0, 6)	N/A	R1: 0	R1: 6	R1: 2	62.5
R2	0.38 (0, 3)	R2	0.75 (0, 3)	N/A	R2: 0	R2: 7	R2:1	(24.5, 91.5)
SUV-early		*PS-late*						
R1	1.25 (0, 5)	R1	1.13 (0, 6)	N/A	R1: 1	R1: 6	R1: 1	75.0
R2	0.75 (0, 3)	R2	0.75 (0, 3)	N/A	R2: 0	R2: 8	R2: 0	(34.9, 96.8)
PS-early		*SUV-late*						
R1	0.25 (0, 1)	R1	1.00 (0, 7)	N/A	R1: 2	R1: 4	R1: 2	37.5
R2	0.38 (0, 3)	R2	0.00 (0, 0)	N/A	R2: 1	R2: 7	R2: 0	(8.5, 75.5)

Abbreviations: Max = maximum; Min = minimum; R1 = reader 1; R2 = reader 2

* Insufficient *n* for precise *P* value calculation but still possible to determine statistical significance

N/A = insufficient cases with different values for V1 and V2 to calculate a *P* value

Note. – The V1 > V2, V1 = V2, and V1 < V2 columns indicate the number of case in which each reader assigned higher, equal, and lower scores to variable 1 relative to variable 2, respectively.
